# Simultaneous Absorption and Desorption Isotherms of Various Hydrogen and Deuterium Mixtures between 20 and 120 °C Are Used to Determine the Activity Coefficients for Palladium-Hydride Solutions

**DOI:** 10.1021/acs.jpcc.4c06976

**Published:** 2025-02-04

**Authors:** Matthew Sharpe

**Affiliations:** Laboratory for Laser Energetics, University of Rochester, 250 East River Road, Rochester, New York 14623, United States

## Abstract

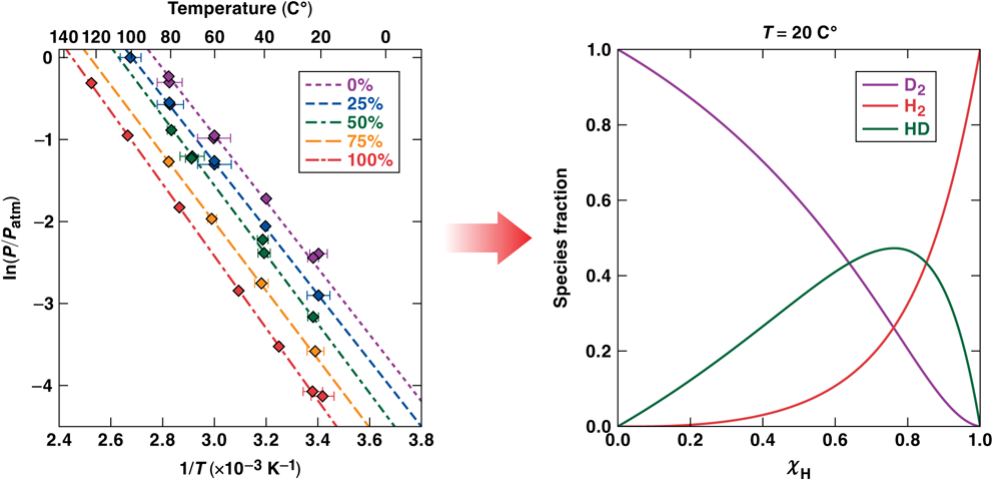

A test bed was constructed to measure the absorption and desorption isotherms for palladium hydride using H_2_, D_2_, and various H_2_/D_2_ mixtures for temperatures in the range 20 °C ≤ *T* ≤ 120 °C. The pressure–composition–temperature isotherms were measured. The pressures obtained with mixtures between each pure isotope were monotonic, yet nonlinear. This nonlinear dependence of total pressure with feed gas protium concentration reveals the mixed isotope hydride system behaves nonideally. A thermodynamic model was adapted from the literature, which accounts for the nonideal nature of the mixed-isotope system. The measured data were used as constraints in this model in order to calculate the protium mole fractions in the hydride phase and the protium activity coefficients for palladium hydride at various temperatures and protium concentrations in the system. Knowing the protium mole fraction and activity coefficients allows for *a priori* calculation of the isotopologue distribution in the gas phase and the isotope distribution in the hydride phase, given a palladium temperature and equilibrium pressure.

## I Introduction

The palladium-hydride system has been studied for many years, with several review articles^1−3^ and many measurements of the pressure–composition–temperature (PCT) isotherms for the pure hydrogen isotopes.^4−9^ This system has received much attention due to the large isotope effect, making it a model system for investigating this effect in metal hydrides in general. Practically, the isotope effect has been exploited for hydrogen isotope separation.^10,11^ Isotope separation processes are becoming increasingly relevant with the recent advances made in nuclear fusion research. Most proposed fusion reactor designs utilize the heavier two hydrogen isotopes, deuterium (D) and tritium (T), as fuel. In these fusion reactions, protium (H) is considered a contaminant. Efficient separation of these hydrogen isotopes from protium is essential to the operation of a fusion reactor by reducing the protium concentrations in the fuel cycle at faster rates. For these reasons, it is essential to understand the fundamental interaction of hydrogen isotopes with palladium, especially in the presence of isotopic mixtures.

The earliest measurements of palladium-hydride systems focused on the PCT absorption and desorption isotherms for the pure isotopes.^4−9^ These measurements were limited to higher temperatures (*T* > 0 °C) and higher pressures. A recent set of measurements by the current author extended the measured PCT isotherms down to much lower temperatures (*T* > −130 °C).^12^ In this work, it was observed that a change in the absorption mechanism appeared for the pure isotopes at temperatures below 236 K for protium and 211 K for deuterium. This change was attributed to the absorption of hydrogen isotopes into subsurface states in the palladium powder; an observation that was consistent with prior reports.^13,14^ While prior work established the trends and hydriding mechanisms trending to low temperatures, it only did so for pure isotopes.

Reported measurements on the mixed isotope systems are limited. Typically, these measurements involve isotope exchange reactions, which are used to quantify the isotope separation factor.^15−18^ The separation factor is defined for the H/D system as
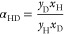
1where *y*_D_ is the deuterium mole fraction in the gas phase, *y*_H_ is the protium mole fraction in the gas phase, *x*_H_ is the protium mole fraction in the solid phase, and *x*_D_ is the deuterium mole fraction in the solid phase. This parameter is useful for benchmarking the equilibrium state of the mixed-isotope system or comparing the extent of the isotopic exchange reaction over palladium; however, it offers a convoluted picture of the isotopic distribution.

The current work adds to the database of information surrounding the Pd-HD system by first showing the measured PCT absorption and desorption isotherms for various H_2_/D_2_ feed-gas mixtures for temperatures between 20 and 120 °C. These data show the full phase space for the Pd-HD system relating to composition, feed-gas isotopic ratio, and temperature (for the measured range). Second, the data from these measurements can then be utilized to determine the activity coefficients and protium mole fractions in the hydride phase using a thermodynamic model. This model predicts the isotopologue fractions for a range of palladium temperatures and protium fractions in the feed gas.

## II Thermodynamics of the Mixed Isotope System

The partial pressures of the various isotopologues can be expressed in terms of the chemical activity of protium in the palladium solution and the isotopic fraction in the palladium. In their paper regarding separation factors of protium and deuterium in palladium, Luo et al.^15^ showed a thermodynamic treatment of the mixed-isotope system. In that paper, the authors started by assuming an equilibrium state between the gas phase and the metal solution. They then derived the following expression for the partial pressure of the pure isotopologues, assuming a nonideal system
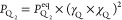
2where *P*_Q_2__ is the partial pressure of a pure isotopologue (H_2_ or D_2_) at equilibrium with palladium for a given temperature, *P*_Q_2__^eq^ is the vapor pressure in the pure isotope system, γ_Q_ is the activity coefficient of the isotope Q, and χ_Q_ is the mole fraction of Q in the palladium. The equilibrium pressure for HD (*P*_HD_) is determined by assuming the equilibrium constant for the formation of HD (*K*_HD_) in the gas phase
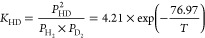
3In the present paper, the equation for the equilibrium constant shown on the right side of eq 3 is an empirical relationship reported by San Marchi et al.^19,20^

In keeping with Luo et al., the activity coefficients for protium and deuterium are assumed to be equal, due to their similar chemical environments. Given this assumption, the partial pressures of each species can be expressed in terms of the protium activity coefficient and mole fraction of protium, as well as the vapor pressures for the pure isotopes
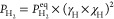
4
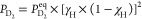
5
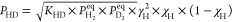
6

These protium activity coefficient and solution mole fractions can be determined by comparing to the measured plateau pressures in the PCT curves for various gas mixtures. To fit the data, two constraints must be applied:

1. Each combination of χ_H_ and γ_H_ yield one total calculated pressure (*P*_H_2__ + *P*_HD_ + *P*_D_2__) which must be equal to the measured value
2. The calculated isotopic distribution in the system (gas phase and metal solution) resulting from the combination of χ_H_ and γ_H_ must yield the overall isotopic distribution in the system set by the experiment

Once the χ_H_ and γ_H_ values are known, the gas-phase partial pressures and the solution-phase isotopic distribution can be calculated.

## III Experimental Setup and Methods

All PCT curves measured in this work utilized a benchtop gas manifold with an attached, single-ended palladium getter bed. The getter bed contained 2.4 g of 99.95% pure palladium sponge with particle diameters ranging from 44 to 149 μm. The palladium packing density was 2 g/cm^3^. The palladium powder used in this study had undergone >10 absorption and desorption cycles prior to isotherm data. Desorption cycles usually involve vacuum baking the powder at 200 °C for several hours. It was observed that after initial conditioning, the isotherms were reproducible after vacuum baking, even after an air exposure.

**Figure 1:**
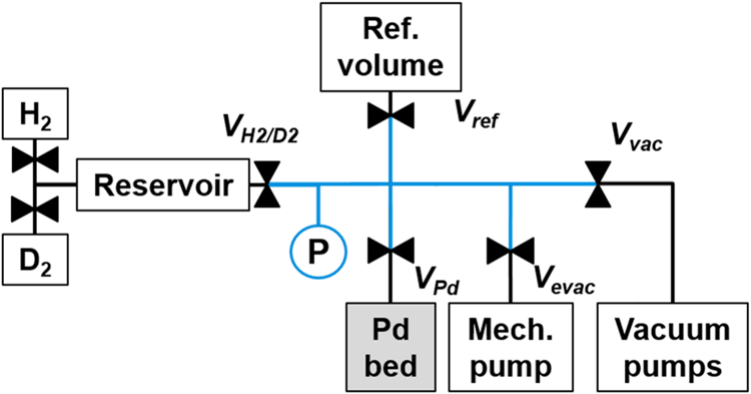
Block diagram of gas manifold and vacuum system used to measure the absorption and desorption isotherms.

A schematic of the setup used is shown in Figure 1. The inlet to the Pd bed is connected to a calibrated gas manifold (shown in blue in Figure 1), which is used to determine the quantity of protium or deuterium added onto the bed. The pressure of gas within this volume is continuously monitored using an MKS 690A Baratron, which has a dynamic range of 0.01 to 105 Pa. This gas manifold is also attached to a reference volume, a reservoir where the H_2_/D_2_ mixtures were created, and two vacuum pumping systems. The reference volume was used to calibrate all volumes in the setup, including the gas manifold, the reservoir volume, and the head space above the Pd bed. Two gas pumping systems were employed: a mechanical pump and a turbo/scroll combination. The turbo and scroll combination allowed for high vacuum pumping down to 5 × 10^–6^ Pa, while the mechanical pump was used for quick evacuation of large quantities of hydrogen isotopes from the gas manifold. The temperature of the palladium was measured using a type-K thermocouple mounted in the stainless-steel container that houses the palladium powder.

Absorption isotherms were measured by sequentially adding gas onto the bed. This was done by pressurizing the reservoir with 99.999% pure protium and deuterium to the desired pressure levels. All initial gas mixture compositions were calculated based on pressure. Aliquots of this gas mixture were then expanded into the gas manifold, then subsequently transferred to the palladium by opening the isolation valve between the gas manifold and the Pd bed (*V*_Pd_). Once an equilibrium pressure was attained, more gas was added by first closing *V*_Pd_ and then repressurizing the gas manifold. *V*_Pd_ was then opened to load the additional gas to the bed. Hydrogen to palladium ratios for each gas addition were calculated using eq 7
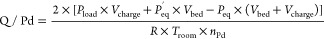
7where *P*_load_ is the charge pressure, *P*_eq_^′^ is the equilibrium pressure after the previous loading, *P*_eq_ is the equilibrium pressure after the current loading, *V*_bed_ is the headspace volume of the palladium bed, *V*_charge_ is the volume of the gas manifold, *n*_Pd_ is the number of moles of palladium, and *T*_room_ is the ambient room temperature. To compute the cumulative hydrogen to metal ratio (Q/Pd), the results of eq 7 were summed after each gas addition. The factor of 2 represents the molar ratio of atomic to molecular hydrogen. Hydrogen was loaded onto the palladium bed in aliquots ranging from 0.1 to 1 mmol H_2_ or D_2_ per loading. After a complete absorption isotherm was measured, either the desorption isotherm was measured or the bed was regenerated by vacuum baking at temperatures at 200 °C. If the desorption isotherm was measured, the bed was regenerated by vacuum baking after desorbing most of the gas.

Desorption isotherms were measured in a similar manner to the absorption isotherms: gas was sequentially removed from the system. Removal of gas was performed by isolating the palladium bed, then evacuating the gas manifold volume. After evacuation, the palladium bed headspace was opened to the now evacuated gas manifold. Due to the pressure decrease on volume expansion, hydrogen isotopes desorbed from the palladium bed. This procedure was repeated until the equilibrium pressure was less than 133 Pa. Hydrogen to metal ratios during the desorption phase were calculated using eq 8
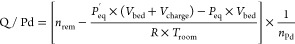
8where *n*_rem_ is the number of moles of hydrogen isotopes remaining on the bed after each desorption; the other terms are the same as in eq 7. Note that *n*_rem_ decreases after each desorption, where *n*_rem_ is computed from the previous desorption using the equation in the brackets in eq 8. This portion of eq 8 represents the difference between the number of moles remaining in the palladium solution (*n*_rem_) and the number of moles desorbed into the gas phase. It is anticipated that the isotope distribution in the system will change as gas is removed from the system. For clarity, all desorption isotherms are referenced by the fraction of protium used in the absorption portion of the experiment.

## IV Results

**Figure 2:**
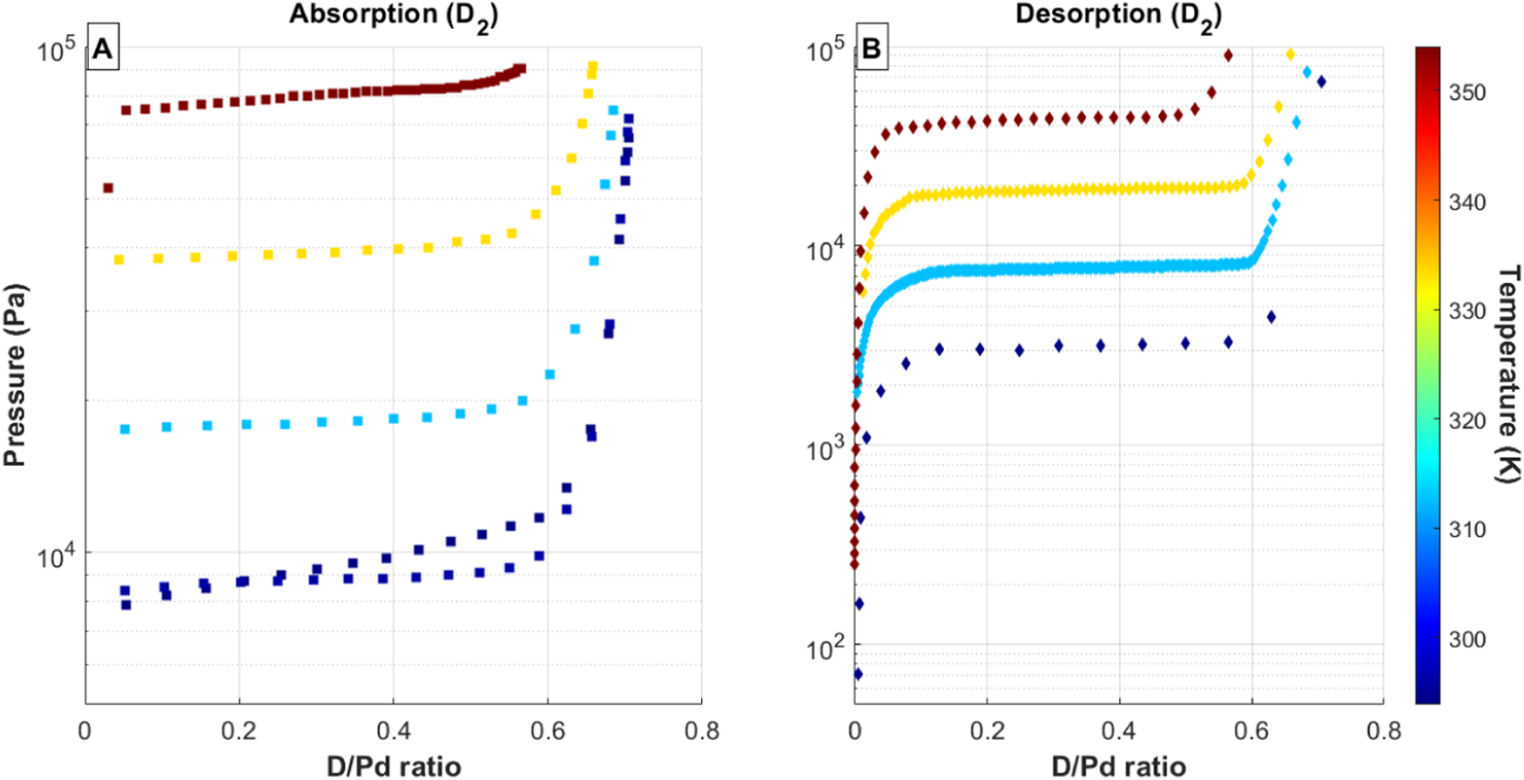
(A) Absorption and (B) desorption PCT curves for 100% D_2_ gas mixtures and Pd bed temperatures ranging from 20 to 80 °C. Data series colors correspond to palladium temperature, with the temperature scale shown as a color bar on the right side of each figure.

**Figure 3:**
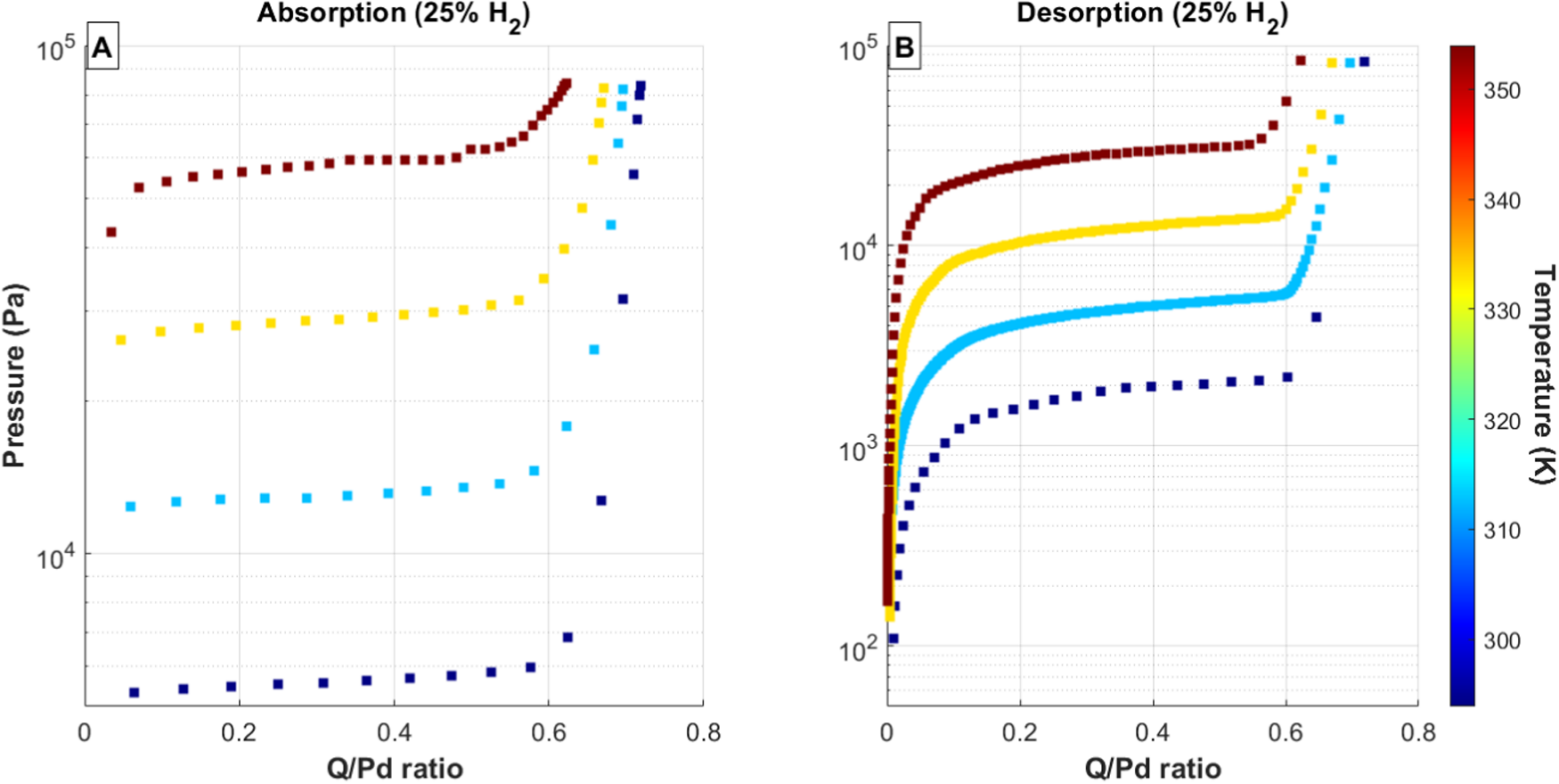
(A) Absorption and (B) desorption PCT curves for 25% H_2_ gas mixtures and Pd bed temperatures ranging from 20 to 80 °C. Data series colors correspond to palladium temperature, with the temperature scale shown as a color bar on the right side of each figure.

**Figure 4:**
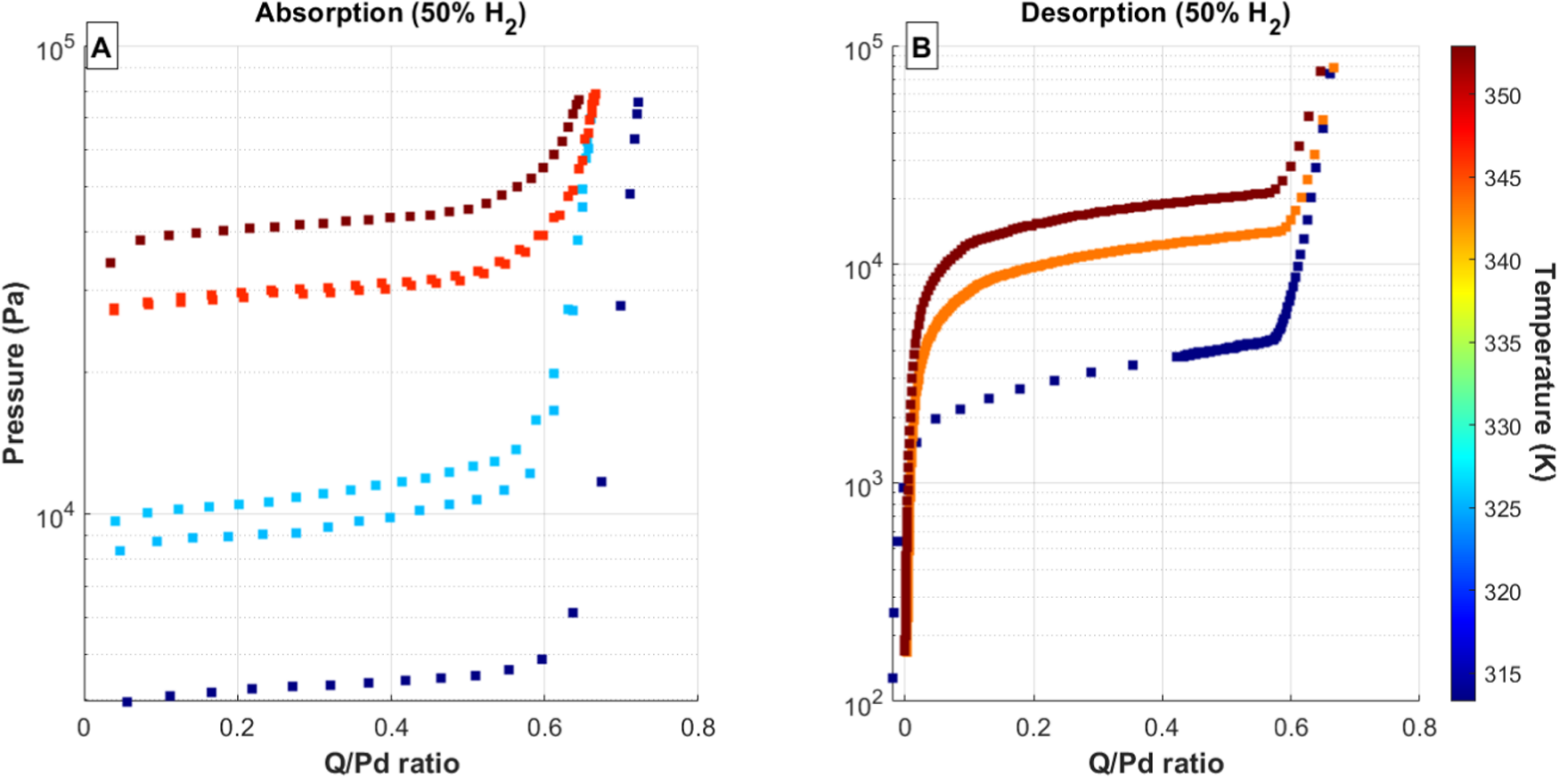
(A) Absorption and (B) desorption PCT curves for 50% H_2_ gas mixtures and Pd bed temperatures ranging from 20 to 80 °C. Data series colors correspond to palladium temperature, with the temperature scale shown as a color bar on the right side of each figure. The desorption curve measured at 40 °C shows increasing spacing between measured data points due to an intentional increase in the desorption volume.

**Figure 5:**
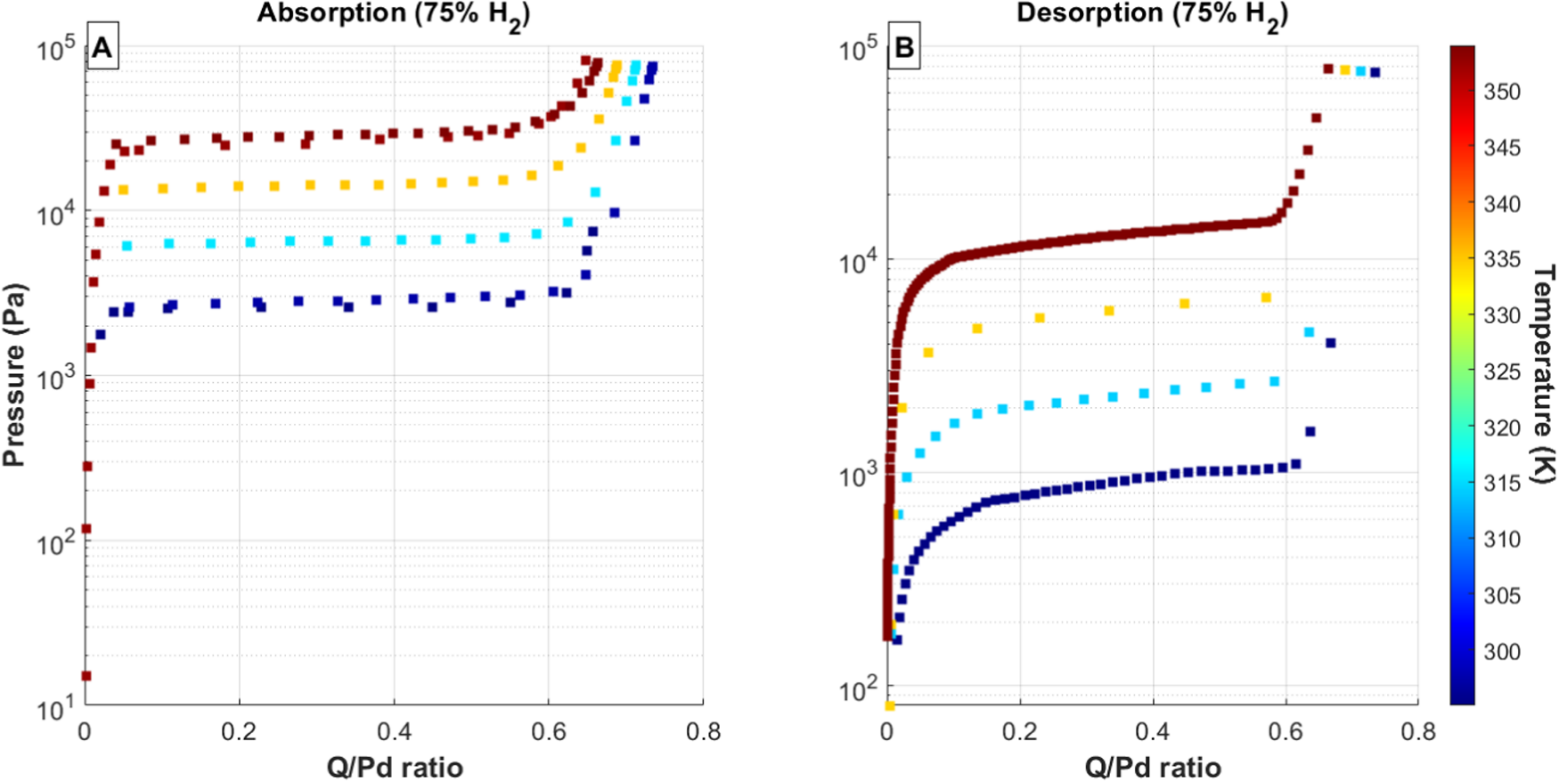
(A) Absorption and (B) desorption PCT curves for 75% H_2_ gas mixtures and Pd bed temperatures ranging from 20 to 80 °C. Data series colors correspond to palladium temperature, with the temperature scale shown as a color bar on the right side of each figure.

**Figure 6:**
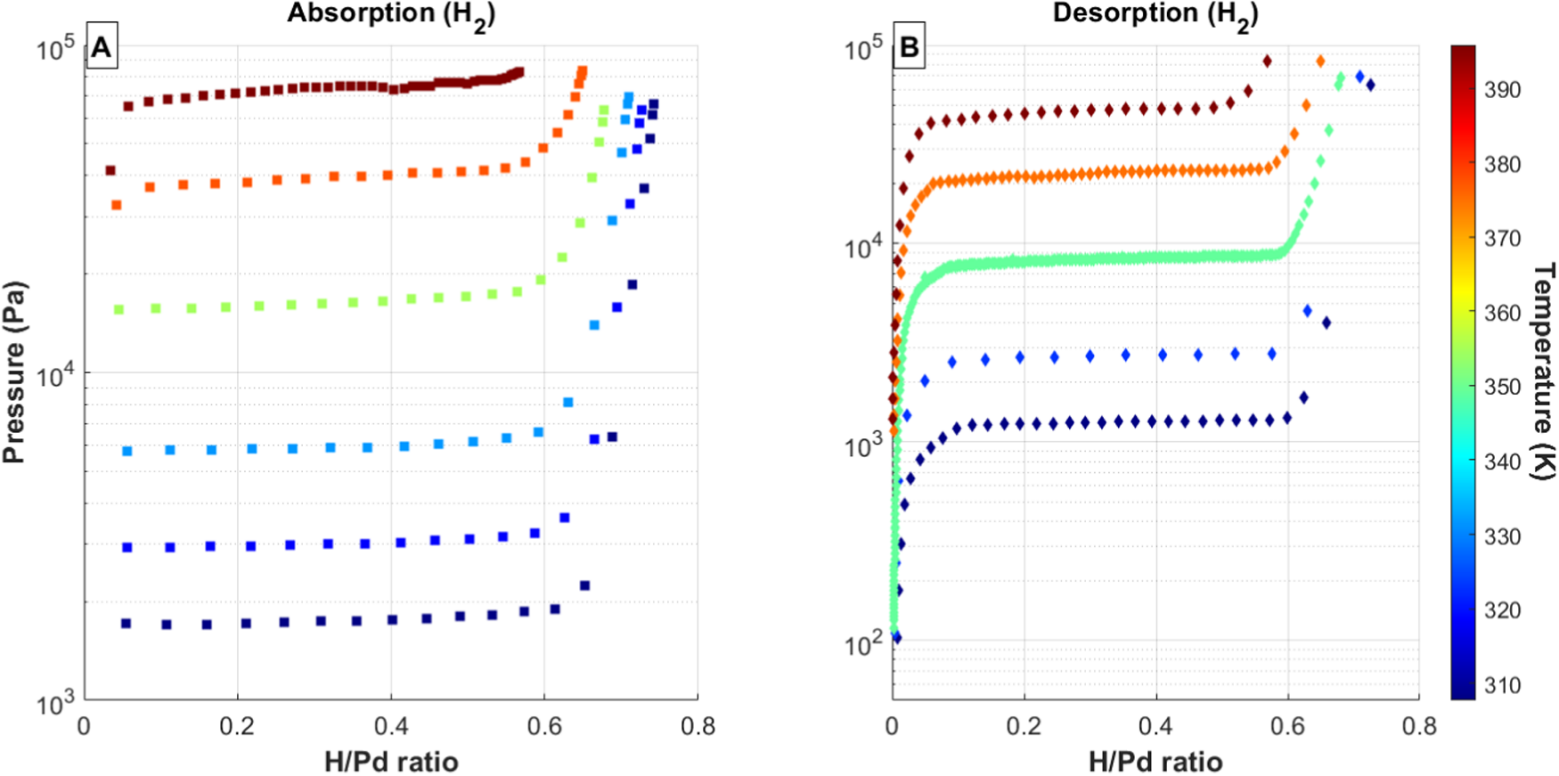
(A) Absorption and (B) desorption PCT curves for 100% H_2_ gas mixtures and Pd bed temperatures ranging from 20 to 120 °C. Data series colors correspond to palladium temperature, with the temperature scale shown as a color bar on the right side of each figure.

Using the above procedure and experimental setup, the absorption and desorption PCT curves were measured for both pure isotopes and three mixtures of H_2_ and D_2_: 25% H_2_, 50% H_2_, and 75% H_2_. For the H/D mixtures, hydrogen and deuterium concentrations in the feed gas were determined by pressure. Each PCT curve was measured by sequentially adding or removing gas from the Pd bed. The results of these measurements are shown in Figures 2, 3, 4, 5, and 6. In each of these figures, the total equilibrium pressure is plotted against the total hydrogen to metal ratio (Q/Pd) for a range of temperatures. For clarity, the absorption and desorption isotherms are split into separate subfigures for each H/D mixture. The color of each data series corresponds to the palladium temperature, with the scale shown as the color bar to the right of each figure. Each PCT curve exhibits the same two-phase palladium-hydride behavior as observed for the pure isotopes. The absorption isotherms exhibit a sharp transition between the α phase and the initial onset of the β phase, while the desorption isotherms show a more gradual change from the mixed α–β region back into the α phase. In many cases, the α region was not measured. This is a result of the experimental method: initial loading of hydrogen isotopes was large enough in these cases that the α region was bypassed. For both the absorption and desorption isotherms, the transition from the mixed α–β plateau to the β phase shows a more-gradual change with increasing hydrogen isotope concentration in the solid. In each case, the maximum total hydrogen to metal ratio decreases with increasing temperature.

**Figure 7:**
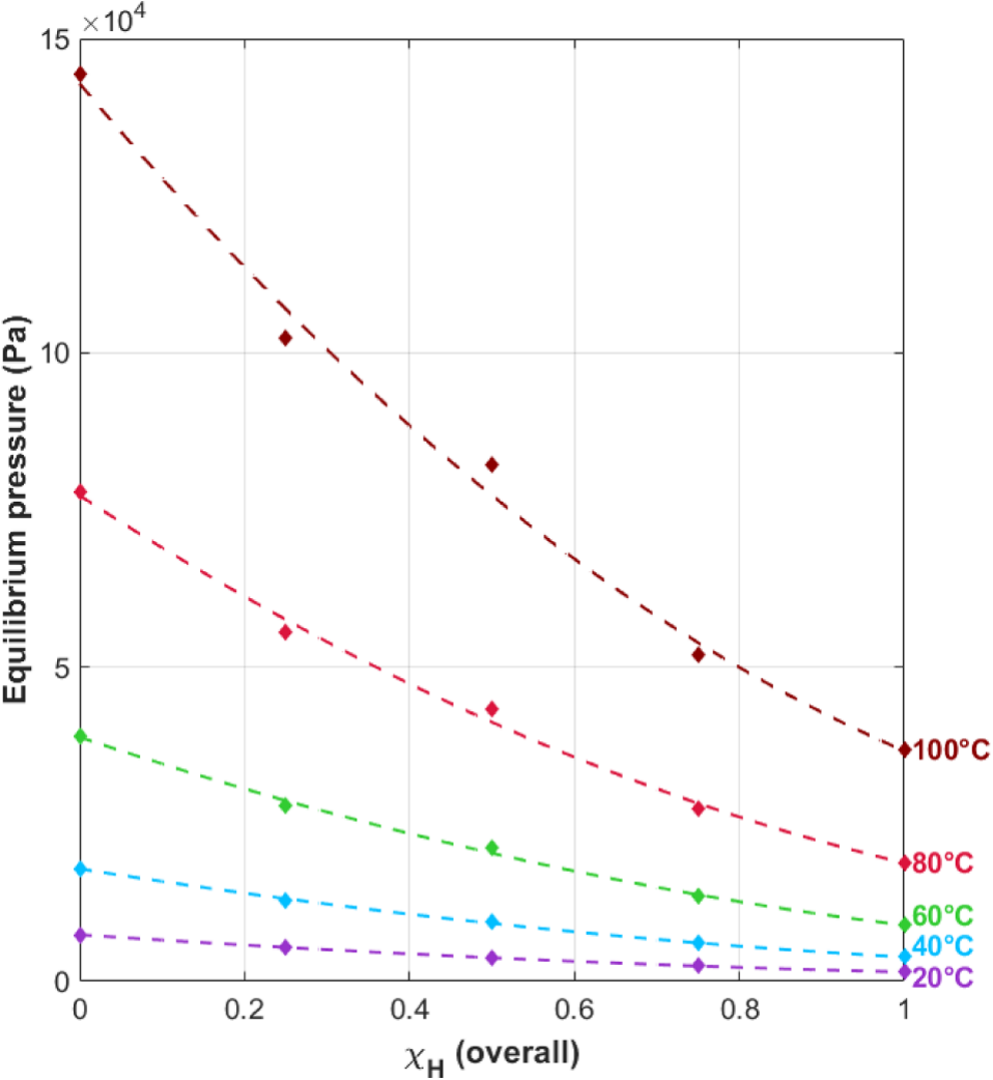
Variation of overall equilibrium pressure with overall protium mole fraction in the system. Data points were calculated from van’t Hoff constants obtained from the absorption isotherms for each temperature and H/D mixtures. Each data series was fit with a second-order polynomial (dashed lines).

All isotherms measured using H/D mixtures show increasing equilibrium pressures with increasing temperature or increasing deuterium concentration in the feed gas. Figure 7 shows the variation of total pressure with temperature and protium mole fraction in the feed gas. Increasing pressures with increasing temperatures is expected from the thermodynamics of the hydride system: the hydriding reaction is exothermic, regardless of which isotope is used. Addition of heat to the system drives the equilibrium toward the reactants, or gas phase, thereby increasing the pressure.

Increasing equilibrium pressures with increasing deuterium content is the result of the large isotope effect observed with palladium hydride: protium more readily forms a hydride than deuterium due to the difference in zero-point energies.^21^ Because of the larger energy difference between gas molecules and absorbed species for lighter isotopes, the resulting equilibrium pressures for pure protium are lower than for pure deuterium. With a H_2_/D_2_ mixture, both protium and deuterium reactions with palladium occur simultaneously. The resulting total pressure at equilibrium is reflective of the extent of each reaction. With increasing deuterium concentrations in the feed gas, the deuterium–palladium reaction becomes increasingly dominant. This results in increasing total pressures at equilibrium, with deuterium being the dominant isotope in the gas phase.

The nonlinear behavior observed for the total pressure with total protium content in the feed gas (Figure 7) indicates the mixed-isotope system is not an ideal system. To demonstrate the nonlinear behavior, the data in Figure 7 were fit with second-order polynomials for each temperature. The resulting fits are shown as dashed lines in the figure. This nonideal behavior is attributed to the formation of the mixed isotope species, HD. Formation of HD couples the protium and deuterium reactions, thereby violating the definition of an ideal system: that is, both solutes react independently from each other.

The desorption isotherms using the mixed H/D feed gases (Figures 3–5) exhibit the normal hysteresis between absorption and desorption pressures in all cases and also show a gradual sloping across the mixed α/β plateau region for the desorption isotherms. This sloping is likely a result of the experimental method shifting the isotopic composition in the system. Each desorption isotherm starts in the β phase, where the dominant gas species is expected to be deuterium. Because the experimental procedure of removes gas sequentially from the system, more deuterium will be removed during the first portion of the desorption isotherm than during the later portion. This would cause a shift of the overall equilibrium pressure toward lower values since the system will become increasingly dominated by protium.

**Figure 8:**
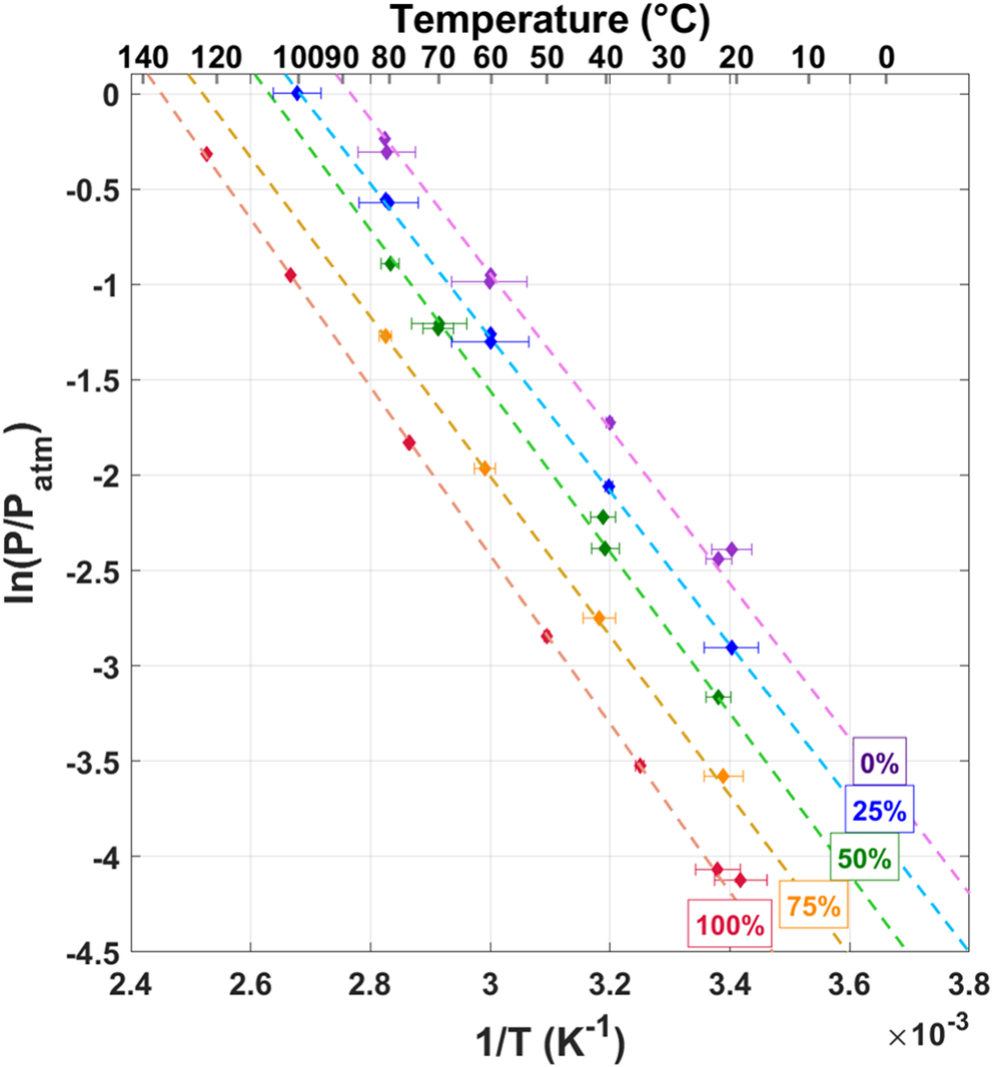
van’t Hoff diagram for the absorption isotherms. All data series fit with a straight line to determine the van’t Hoff constants. Mixtures are represented by five different colors: red (100% H_2_), orange (75% H_2_), green (50% H_2_), blue (25% H_2_), purple (100% D_2_).

**Figure 9:**
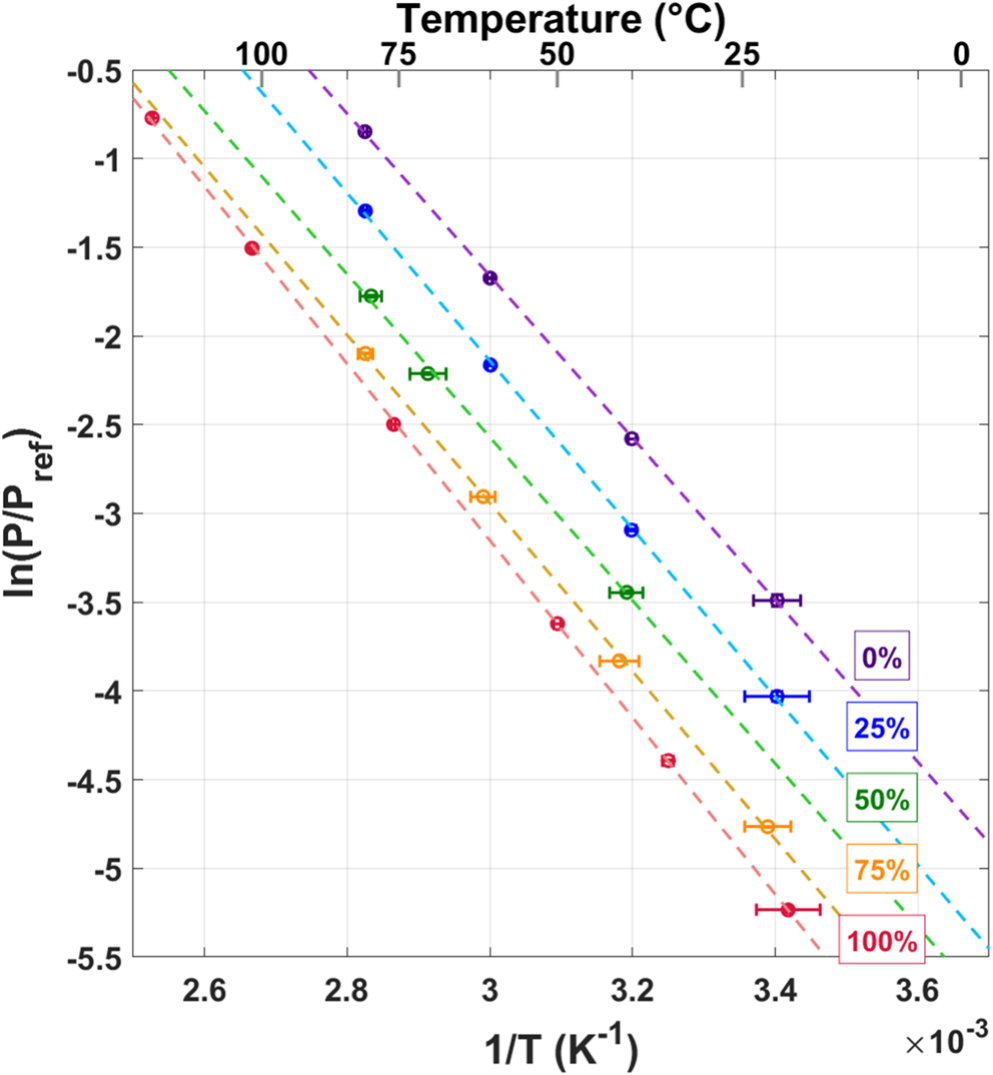
van’t Hoff diagram for the desorption isotherms. Mixtures are represented by five different colors: red (100% H_2_), orange (75% H_2_), green (50% H_2_), blue (25% H_2_), purple (100% D_2_). All data series fit with a straight line to determine the van’t Hoff constants.

Two van’t Hoff plots were constructed to show the dependence of the total equilibrium pressure on temperature for the various feed gas mixtures. These plots were constructed using a similar method as outlined in a previous publication.^12^ Briefly, the equilibrium pressures are plotted as a function of the inverse temperature. The resulting straight line can be fit with
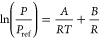
9where *P* is the equilibrium pressure, *P*_ref_ is the reference state pressure (1 atm), *T* is temperature in Kelvin, *R* is the gas constant, and *A* and *B* are the van’t Hoff constants. For the current work, measured equilibrium pressures were taken at a total hydrogen to metal ratio of 0.3, which is in the middle of the mixed α–β plateau region. Values for the equilibrium pressure were determined by fitting a line to the data between Q/Pd = 0.2 and Q/Pd = 0.4. Equilibrium pressures at Q/Pd = 0.3 were calculated from this best fit line. Figure 8 shows the resulting van’t Hoff plots for the absorption isotherms while Figure 9 shows the desorption data. Where applicable, previously reported data for pure isotopes are also included for reference.^4−9^ The results for the mixed gases show linear trends in the van’t Hoff figure, which are roughly parallel to the pure isotopes, yet span the region between the pure isotope data. Fitting the results for each gas composition with a linear trend yields the van’t Hoff constants (Table 1).

**Table 1:** Absorption and Desorption van’t Hoff Constants for H_2_/D_2_ Gas Mixtures^a^

% H_2_	*A*_abs_ (kJ/mol H_2_)	*B*_abs_ (J/K)	*A*_des_ (kJ/mol H_2_)	*B*_des_ (J/K)
0	–36.6	89.8	–41.4	98
25	–34.7	87.5	–39.33	93.5
50	–35.1	92.3	–38.2	93.2
75	–33.5	89.8	–39.3	100
100	–33.7	93.3	–37.9	100

a Constants reported in this table were calculated by multiplying the slope and intercept by the gas constant (*R*).

## V Discussion

**Figure 10:**
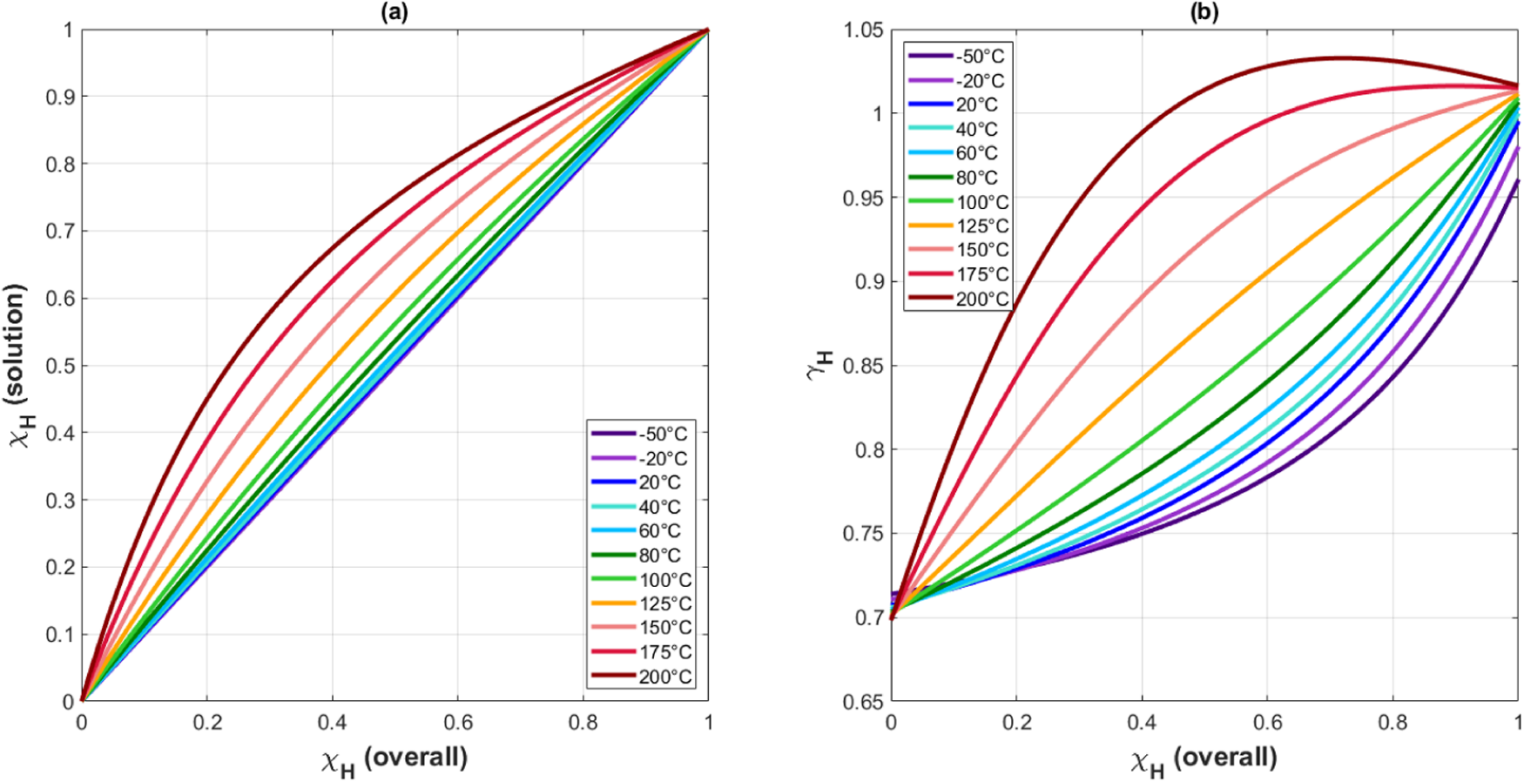
(a) Calculated protium mole fraction in the metal solution and (b) the associated activity coefficients as a function of the total protium mole fraction in the feed gas. The mole fractions and activity coefficients were calculated for a range of temperatures, all spanning the linear range in the van’t Hoff figure (Figure 8).^12^

**Figure 11:**
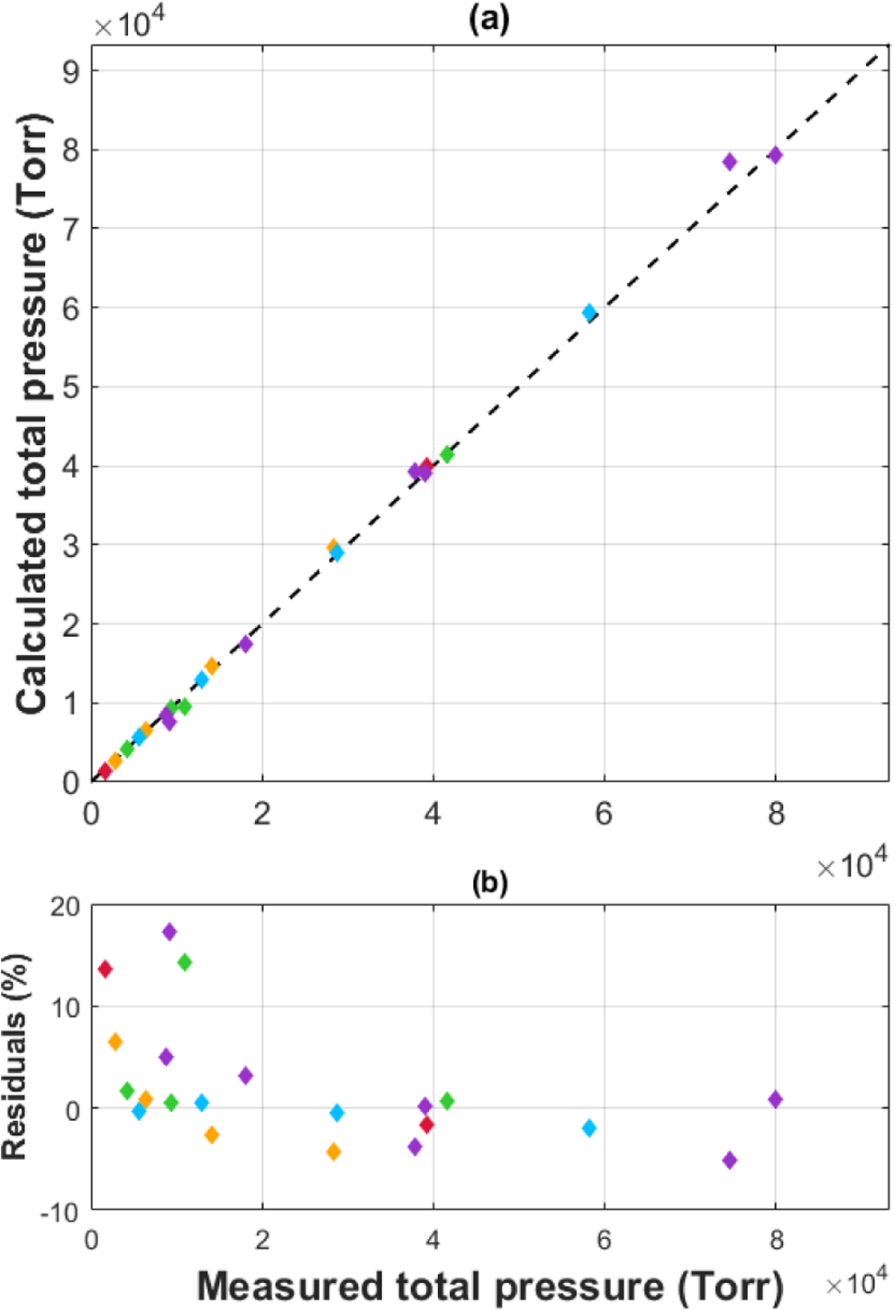
(a) Comparison between the calculated and measured equilibrium pressures for various feed gas mixtures: 100% H_2_ (red), 75% H_2_ (orange), 50% H_2_ (green), 25% H_2_ (blue), and 100% D_2_ (purple). A representative correlation line (dashed black line) is included for reference. (b) The deviation between the calculated and measured pressures for each feed-gas mixture.

Using the equilibrium absorption pressures measured in the mixed α/β region for the various H_2_/D_2_ feed-gas mixtures, the protium mole fractions in the hydride solution and the associated protium activity coefficients were determined by fitting the data as outlined in Section II. In order to determine the mole fractions and activities for all feed-gas mixtures, the second-order polynomial fits to the measured data were used (Figure 7). The results of these fits are shown in Figure 10. Here, the protium mole fractions [Figure 10a] and the activity coefficients [Figure 10b] are shown for a range of temperatures and total protium fractions in the feed gas. The temperatures chosen span the linear region in the van’t Hoff plots, which correspond to the hydriding region.^12^ As a check of the calculation, Figure 11 shows the comparison between the measured and calculated total pressures, with a linear correlation line for reference. These residuals show a mean overall deviation of 1.7% and a maximum of 12.9%. Major sources of error in the calculation may stem from utilizing multiple fits to the data. First, the calculation relies on using the van’t Hoff parameters (Table 1) to calculate equilibrium pressures for a given mixture at a particular temperature. Second, a second-order polynomial was used to determine the equilibrium temperature for all feed-gas mixtures. Each of these fits will cause a deviation between the calculated and measured pressures. Additionally, there are errors in the data that will contribute to the deviation.

The protium mole fractions show that as temperature decreases, the calculated mole fraction in solution trends toward being equal to the mole fraction in the feed gas (Figure 10). This trend is a result increasing absorption at lower temperatures. Lower temperatures will result in increased absorption due to the exothermic nature of the hydriding reaction (Figures 2–6). Increasing absorption will cause the isotopic fractions in the hydride solution to become closer to the feed-gas fractions, since most of the gas will be absorbed.

On the other hand, increasing the temperature results in higher protium fractions in the hydride solution for a given feed-gas concentration. Higher protium fractions indicate that more deuterium remains in the gas phase during absorption from a mixed feed gas. This result is in agreement with both the known energetics of the system as well as the reported isotope separation factors. First, it is known that the Pd–H bond is stronger than the Pd–D bond. This difference is observed as a higher reaction enthalpy for Pd + H_2_ (Table 1) and has also been reported in thermal desorption spectroscopy studies.^14,21^ From these data, it is expected that deuterium will favor the gas phase to a greater degree than protium as the temperature is increased. Second, the reported isotope separation factors are greater than unity for all temperatures and palladium phases.^15−18^ Separation factors greater than unity indicate more deuterium is present in the gas phase than in solution relative to the same ratio for protium. Putting all these data together, one should expect that as the temperature is raised, both protium and deuterium are released into the gas phase from the Pd–H_*x*_D_*y*_ solution. However, a greater fraction of gaseous deuterium will be present than gaseous protium due to the above effects. While the present calculations may seem to suggest an increasing isotope effect at greater temperatures, this is not necessarily the case. Indeed, the isotope effect decreases at lower temperatures; the ratio of equilibrium pressures for pure protium and pure deuterium show this to be the case. The competing effects of increased absorption at lower temperatures and the presence of the isotope effect at higher temperatures can lead to the justifiable calculated results above: an increase in protium fractions in solution at higher temperatures.

**Figure 12:**
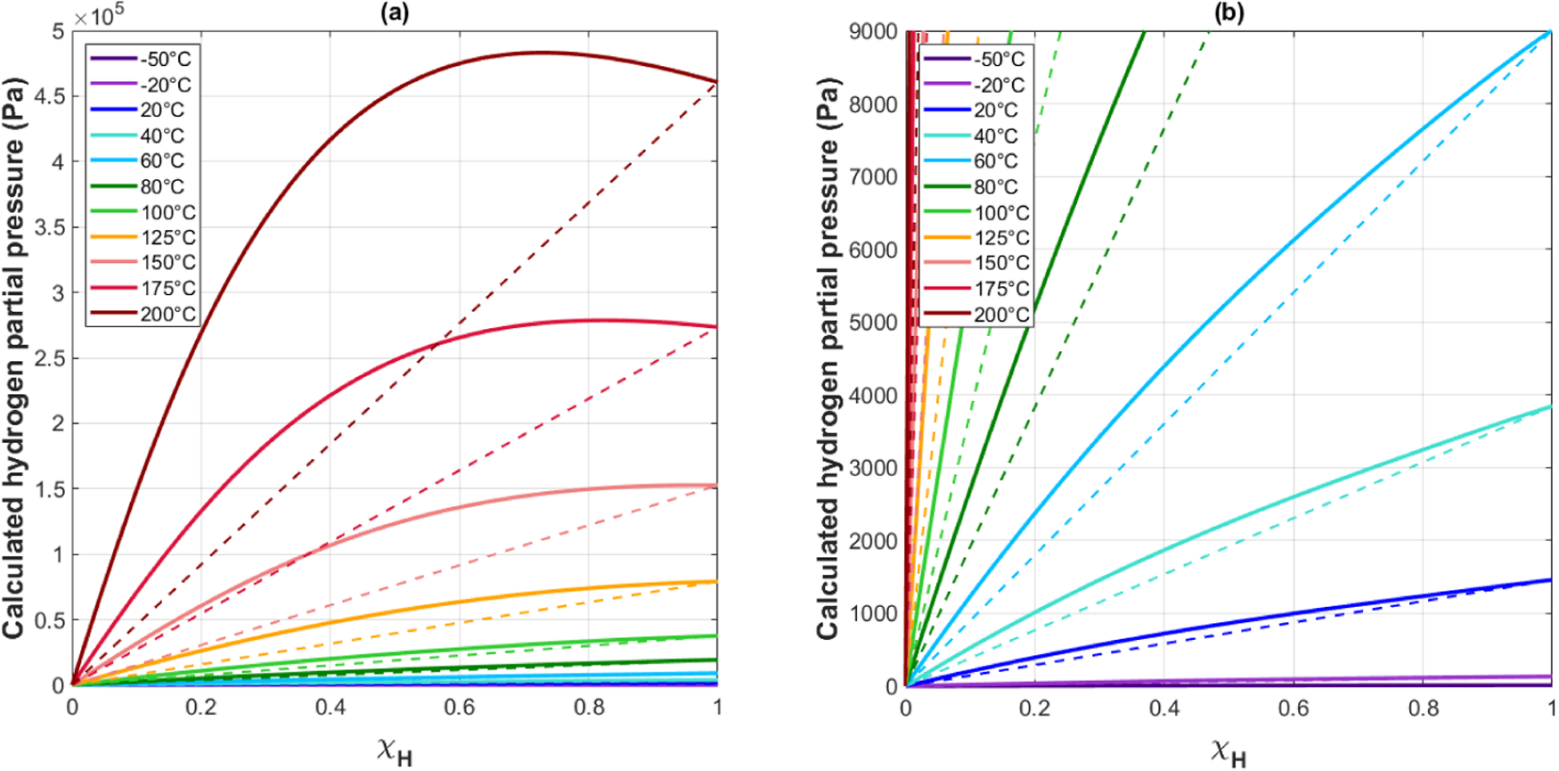
(a) Calculated hydrogen partial pressure for various mole fractions of hydrogen in the feed gas. These partial pressures were calculated using the hydride mole fractions and activity coefficients shown in Figure 10. Expected ideal response shown for each temperature are shown as dashed lines. (b) Plot is a magnified view of the lower pressure range shown in (a).

The activity coefficients resulting from the fit to the data show distinctly nonideal behavior (γ ≠ 1) for all temperatures and feed-gas mixtures (Figure 10). Nonideal behavior is expected for the mixed isotope system due to the formation of the interisotope species, HD. During absorption of H_2_ and D_2_, HD is expected to form due to a dynamic equilibrium between the gas and solution phases. HD formation links the Pd–H and Pd–D reactions, thereby violating the primary condition necessary for ideal behavior of noninteracting species. This nonideal behavior is observed as a deflection from the ideal case for all temperatures when plotting the hydrogen partial pressures as a function of the overall protium fraction in the feed gas (Figures 7 and 12). Hydrogen partial pressures were calculated by
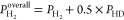
10where *P*_H_2__ is the protium partial pressure, *P*_HD_ is the mixed isotope partial pressure, and *P*_H_2__^overall^ is the overall hydrogen partial pressure including the HD response. The linear relations expected for an ideal system are included in Figure 12 for reference. All hydrogen partial pressure trends show positive deflections relative to the ideal case. Positive deflections typically correspond to activity coefficients less than unity for the trends observed. The calculated hydrogen pressures show a maximum that develops at *T* ∼ 155 °C (not shown for clarity) and shifts to lower protium mole fractions with higher temperatures. At 200 °C, the maximum shifts to χ_H_ ∼ 0.75. The presence of these maxima may be the cause of the activity coefficients exceeding unity for the higher temperatures. The presence of the mixed isotope species convolutes this interpretation, however, since the system is not a binary mixture. Additionally, due to limitations of the current experimental setup (pressure gauge maximum = 1000 Torr), this temperature range could not be investigated in the current study. Future measurements may elucidate the high-temperature trends.

**Figure 13:**
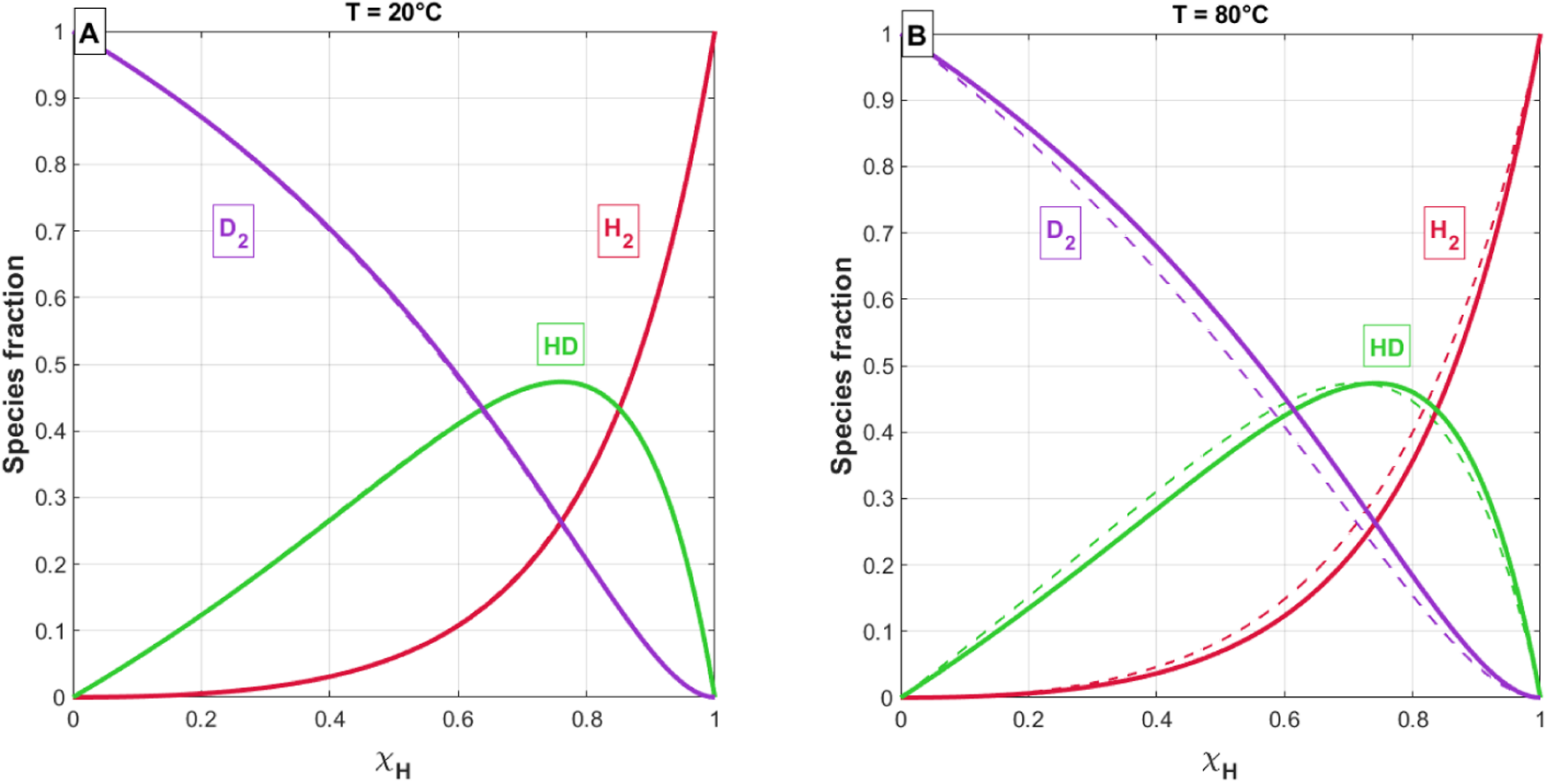
Calculated gas phase isotopologue fractions for Pd temperatures of (A) 20 °C and (B) 80 °C. These fractions are plotted against the hydrogen fraction in the feed gas (dashed lines) and against the fraction of hydrogen in solution (solid lines). Isotopologue fractions were calculated using the absorption isotherm pressures given by the van’t Hoff constants (Table 1) and the quadratic fits to the total equilibrium pressures for various overall hydrogen fractions (Figure 7).

Inserting the protium mole fractions and activity coefficients shown in Figure 10 into eqs 4–6, the isotopologue fractions in the gas phase were calculated for palladium held at 20 and 80 °C (Figure 13). Each fraction is plotted against the hydrogen fraction in the feed gas (dashed lines) and the hydrogen fractions in the Pd metal (solid lines). The same methodology can be used to determine the parameters resulting from desorption isotherms and/or palladium-hydride phase (i.e., α, β, or mixed).

The calculated gas-phase isotopologue fractions show a distribution that is dominated by deuterium for most feed-gas mixtures and does not significantly change with temperature. It is perhaps no surprise that deuterium remains the dominant species in the gas phase due to its lower affinity for forming a hydride. The distribution does not change significantly with pressure because of the roughly parallel response observed in the van’t Hoff diagrams for all HD mixtures (Figures 8 and 9). The agreement at 20 °C in Figure 13A is a result of increased hydride formation at lower temperatures. At lower temperatures, most of the hydrogen isotopes are absorbed in the solid phase. Therefore, the protium mole fraction in the hydride phase is increasingly reflective of the overall protium fraction in the system.

## VI Conclusions

The absorption and desorption isotherms for H_2_, D_2_, and various H_2_/D_2_ mixtures were measured for temperatures ranging from 20 to 120 °C. The pure isotope responses agreed favorably with reported values. No such data were available for comparison of the current H_2_/D_2_ mixtures, making these potentially the first reported isotherms. These isotherms showed equilibrium pressures that spanned the region between the pure isotope responses in a nonlinear fashion. This nonlinear behavior indicates the mixed isotope system is a nonideal system. For temperatures up to 100 °C, this nonideal behavior shows a negative deviation relative to the ideal Raoult’s law.

Using the isotherm data as constraints for a thermodynamic model allows for the calculation of the protium mole fractions in the palladium solution and protium mole fractions for various H_2_/D_2_ mixtures and palladium temperatures. The resulting activity coefficients were less than unity for temperatures less than 100 °C, in agreement with expectations from the data. The protium mole fractions showed that as temperature decreased, the mole fractions in solution agreed better with the feed-gas mole fractions. This trend is a result of increased absorption at lower temperatures: with more gas being absorbed, the solution phase will more-accurately reflect the feed-gas concentrations. At higher temperatures, the calculated protium fractions in the palladium solution increased. Higher fractions at these temperatures suggest more deuterium remains in the gas phase during absorption. A future set of experiments is planned to include measurements of the gas phase isotopologue distributions. Data resulting from these experiments can then be compared against the results shown here.

The thermodynamic model using in this work allows for the use of these protium mole fractions and activity coefficients to predict the gas phase isotopologue and hydride phase isotope distributions, given only the total pressure and palladium temperature. This can be utilized practically for modeling hydrogen isotope separation processes that use palladium as a basis. While the focus of this paper was on the absorption data in the mixed α/β phase, the same analysis can be carried out for the pure α- and β-palladium hydride phases.
